# Full-Scale Multistage Pressure-Retarded Osmosis for Osmotic Energy Generation: Optimization of Specific and Net Energy Production

**DOI:** 10.3390/membranes16090299

**Published:** 2026-09-12

**Authors:** Daniel Suárez-Alfonso, Alejandro Ruiz-García

**Affiliations:** Department of Electronic Engineering and Automation, University of Las Palmas de Gran Canaria, Campus de Tafira, E-35017 Las Palmas de Gran Canaria, Spain; daniel.alfonso@ulpgc.es

**Keywords:** pressure-retarded osmosis, blue energy, multistage system, optimization, hollow fiber, specific energy generation

## Abstract

Salinity gradient energy, also known as blue energy, is a clean and renewable option for electricity generation with no direct CO_2_ emissions. Among the available technologies, pressure-retarded osmosis (PRO) stands out, although its large-scale implementation is not yet economically viable, so predictive models are essential to assess its real potential. In the present work, a multistage PRO system of up to three stages, with one–three hollow-fiber membrane modules (HFMMs) arranged in series per stage, was simulated and the operating conditions were optimized. The model accounts for axial pressure drops, feed concentration and draw dilution along each module, considering two salinity gradients of 29.5 and 59.5 g L^−1^. The maximum net specific energy generation reached 421.48 Wh m^−3^ with three stages under the 59.5 g L^−1^ gradient. Adding HFMMs in series benefited the two-stage system but slightly penalized the three-stage one, and the net specific energy decreased monotonically as the ratio of pressure vessels between the first and the second stage increased. At least three stages are therefore required for a full-scale PRO system to become a net energy producer at moderate salinity gradients, and further energy should be sought through additional staging rather than through longer series.

## 1. Introduction

The challenge of generating electricity while minimizing its environmental impact remains one of the key stakes that humankind face [1]. The use of renewable energy sources is becoming increasingly widespread, as is the exploration of new sources [2]. In this regard, electricity generation using salinity gradients (SGs) has been gaining attention over the past two decades [3]. A recent report, prepared for the European Commission’s Directorate-General for Energy, identifies salinity gradient energy, also referred to as osmotic or blue energy, as a clean and renewable energy option in the EU, noting that it can be allocated to hydropower. Moreover, osmotic technologies are highlighted as a promising renewable alternative, capable of providing stable electricity with no direct CO_2_ emissions [4,5]. Among the possible technologies that enable such generation, reverse electrodialysis (RED) and pressure-retarded osmosis (PRO) stand out [6,7]. This is likely because these processes are very similar to those already established for desalinating seawater and brackish water. Significant efforts are being made to improve these technologies for large-scale use, either as massive power generation systems or as systems integrated with other processes to enhance their energy efficiency [8]. This includes the development of more efficient membranes [9] and the incorporation of PRO or RED systems into processes such as RO and other desalination processes [10,11,12]. Unfortunately, both RED and PRO technologies are still in the research phase, and their large-scale implementation is not economically viable. At this stage, predictive models are needed to simulate and optimize these systems at larger scales [13,14]. The main limitations can then be identified, and the required improvements can be determined. This is a key step toward making the technology viable. In many studies, energy production in PRO systems has been estimated, optimal operating conditions have been identified, and the influence of operating parameters on performance has been evaluated.

Based on their configuration, the main membranes being studied for use in PRO systems are flat-sheet membranes (which could potentially be used in spiral-wound modules for large-scale applications) and hollow-fiber membranes [15]. The main advantage of hollow-fiber membrane modules (HFMMs) is that their packing density is roughly one order of magnitude higher than that of spiral-wound membrane modules (SWMM) of equivalent sizes, which reduces the number of PVs required for a given membrane area and is therefore particularly relevant for full-scale PRO systems, where the installed area governs both the energy recovered and the capital cost. Studies related to the manufacture of these membranes typically focus on maximizing power density (*PD*) and ensuring fouling resistance. This is common, as membranes are often manufactured in relatively small sizes for testing and performance evaluation. Typically, when studies are conducted assuming large-scale PRO systems, the *PD* tends to decrease due to the consideration of large-scale operating conditions. This is primarily due to the consideration of pressure drops on both sides (draw side and feed side), axial variations in concentrations, both in the draw (dilution along the membrane element) and in the feed (concentration along the membrane element) and fouling impact estimation on PRO [16,17]. Taking this into account is key to estimating the actual performance that could be achieved with large-scale PRO systems. Computational fluid dynamics (CFD) is increasingly being used to improve the performance of pressure-retarded osmosis (PRO) systems. In particular, CFD studies are being conducted to optimize spacer geometries, with the aim of enhancing mass transfer while reducing concentration polarization and pressure losses within the membrane channels [18]. These models also allow the local interaction between fluid flow, solute transport, and osmotic water flux to be analyzed in complex three-dimensional geometries [19]. Moreover, an accurate description of reverse solute flux and concentration polarization is important for predicting PRO performance, especially in spacer-filled channels [14]. These CFD-based approaches can therefore support the design of more efficient PRO modules for salinity-gradient power generation. Unfortunately, CFD studies cannot be applied to large-scale PRO systems due to their high computational time. Table 1 shows studies related to PRO and records whether these factors have been taken into account, as well as detailing the system configurations (membrane elements in series, multistage processes, etc.) and concentration gradients (salinity difference between the draw and feed solutions at the inlet of the first membrane element) considered. In the few cases, in which the number of stages was varied, the total membrane area was kept constant and redistributed among the stages, so that the effect of adding a stage cannot be separated from the effect of rearranging the existing area. Furthermore, the energy performance has been evaluated at system level only, which conceals how energy is generated and consumed in each individual stage. Finally, the distribution of PVs between consecutive stages has not been treated as a design variable, despite being one of the few degrees of freedom available to a plant designer once the membrane and the salinity gradient are fixed.

As can be seen in Table 1, many studies do not consider multistage configurations, and others do not account for axial variations in pressure and concentrations on both the draw side and the feed side. The main objective of this study is the simulation and optimization of full-scale PRO systems with up to three stages, using one–three HFMMs per stage to verify whether such PRO systems are viable from the perspective of electricity generation, and considering two approximate salinity gradients of 30 and 60 g L^−1^. These salinity gradients were chosen because they are typical between seawater and river water, or between river water and brine from a desalination plant, or between effluent from a wastewater treatment plant and seawater or brine. Both the operating conditions and the PV distributions between stages were optimized to maximize the net specific energy generation (${\mathrm{SEG}}_{\mathrm{net}}$), and the resulting energy balance was resolved stage by stage.

## 2. Materials and Methods

### 2.1. Mathematical Model

The set of equations used in this study to estimate the performance of PRO systems has been previously published by the authors [33,46]. The performance of the PRO process mainly depends on flows (*Q*), pressures (*p*) and concentrations (*C*), along with the membrane characteristics. These characteristics are the water permeability coefficient (*A*), the solute permeability coefficient (*B*) and the structural parameter ($S_{f}$). It should be noted that, in the algorithm used in [33], the membrane configuration considered was SWMM and single-stage, but in [46], the algorithm was adapted to HFMM and single-stage. The equations used are applied considering the average values per HFMM.

The systems were simulated as follows: the output parameters of one HFMM are the input parameters of the next HFMM arranged in series in a PV; within the same stage, the operation in each PV is similar. Also, at each stage, a number of parallel PVs was considered, whose flows were utilized and feed into the next stage. Regardless of these considerations, the pressure drop (Δp) along the HFMM on the draw and feed sides, as well as the feed concentration and draw dilution along the membrane, were taken into consideration.

The fundamental equations of the PRO process is as follows:

(1) $$J_{p}=A\left(\Delta \pi -\Delta p\right)$$

where $J_{p}$ is the permeate flux, Δπ is the osmotic pressure gradient and Δp is the pressure gradient. To calculate the permeate flow rate ($Q_{p}$), $J_{p}$ is multiplied by the membrane surface ($S_{m}$). To determine Δπ, the concentration on the membrane surface needs to be estimated for both the draw and feed sides. To do so, consideration must be given to the effect of the external and internal concentration polarization (ECP and ICP, respectively) [47]:

(2) $$A=A_{0}\;\cdot \;\mathrm{TCF}\;\cdot \;\mathrm{FF}$$

(3) $$\Delta \pi =\pi_{D,m}-\pi_{F,m}$$

(4) $$\pi =3.805C^{2}+42.527C+0.434$$

where $A_{0}$ is the initial value of *A*, TCF is the temperature correction factor [48] and FF is the fouling factor, which has a value of 1 if no fouling is considered. $\pi_{D,m}$ and $\pi_{F,m}$ are the membrane surface osmotic pressures of the draw and feed side, respectively. The purpose of Equation (4) [49] is to obtain the osmotic pressure from the NaCl concentration (in mol L^−1^). To calculate $\pi_{D,m}$ and $\pi_{F,m}$, $C_{D,m}$ and $C_{F,m}$ are used in Equation (4).

The following equations, originally developed for HFMM geometries, replace those used for SWMM in [33]:

(5) $${\mathrm{Sh}}_{D}=0.048\;\cdot \;Re_{D}^{0.6}\;\cdot \;{\mathrm{Sc}}_{D}^{\frac{1}{3}}$$

(6) $${\mathrm{Sh}}_{F}=0.048\;\cdot \;Re_{F}^{0.6}\;\cdot \;{\mathrm{Sc}}_{F}^{\frac{1}{3}}$$

(7) $${\mathrm{PL}}_{D}=\frac{32\;\cdot \;\mu_{D,\mathrm{av}}\;\cdot \;v_{D,\mathrm{av}}}{d_{i}^{2}}L$$

(8) $${\mathrm{PL}}_{F}=\frac{32\;\cdot \;\mu_{F,\mathrm{av}}\;\cdot \;v_{F,\mathrm{av}}}{D_{i}^{2}}L+\frac{150\;\cdot \;{\left(1-\varepsilon_{D}\right)}^{2}\;\cdot \;\mu_{F,\mathrm{av}}\;\cdot \;v_{F,\mathrm{av}}}{1.5^{2}\;\cdot \;\varepsilon_{D}^{3}\;\cdot \;d_{h,F}^{2}}\left(R_{o}-R_{i}\right)+\frac{1.75\;\cdot \;\left(1-\varepsilon_{D}\right)\;\cdot \;\rho_{F,\mathrm{av}}\;\cdot \;v_{F,\mathrm{av}}^{2}}{1.5\;\cdot \;\varepsilon_{D}^{3}\;\cdot \;d_{h,F}}\left(R_{o}-R_{i}\right)$$

where Re is the Reynolds number, Sc is the Schmidt number and Sh is the Sherwood number. PL is the pressure loss, ε is the porosity and $d_{h}$ is the hydraulic diameter of each channel. $R_{i}$ is the inner radius of the fiber bundle ($D_{i}$ is diameter), $R_{o}$ is the outer radius of the fiber bundle and $d_{i}$ is the inner fiber diameter (m). *v* is velocity, ρ is density and μ is the dynamic viscosity.

The rest of the equations from the applied mathematical model can be found in Appendix A.

### 2.2. Simulation Conditions for the Multiple Stages of PRO

The multistage PRO system presented in this study operates on an industrial scale, so a wide range of feed and draw flow rates and pressures were considered. The ranges in Table 2 were established according to the operating envelope of full-scale membrane trains. The flow rates correspond to those typically circulated through a PV in full-scale RO systems. The maximum of 16 m^3^ h^−1^ was set for the $Q_{D,\mathrm{out}}$ and $Q_{F,\mathrm{out}}$, leaving each stage as set. The range of $p_{D,\mathrm{in}}$ spans values both below and above Δπ/2 for the two salinity gradients considered, so that the optimum is not constrained by the search domain. The range of *Y* covers the tapered arrangements normally adopted in full-scale trains, in which the number of PVs decreases from one stage to the next. Limitations in terms of maximum flux recovery (*R*) and $Q_{p}$ per HFMM and per stage were not established. All calculations were performed considering up to 3 HFMMs arranged in series in each PV of every stage. The decision to limit the number of HFMMs in series to 3 was due to its large active surface area: the higher the permeate production, the lower the FS output, *Q*. When considering 4 membrane modules in series, it was observed that very few operating points in the first stage were chosen, as the minimum outflow was not met. Finally, the characteristics of the selected HFMM are shown in Table 3. The values of $A_{0}$, *B* and $S_{f}$ were obtained and validated through forward osmosis simulation tests using the information of the datasheet provided by Toyobo (a first validation approximation was done in [46], but only for $A_{0}$).

### 2.3. Equations for Performance Evaluation

**Figure 1:**
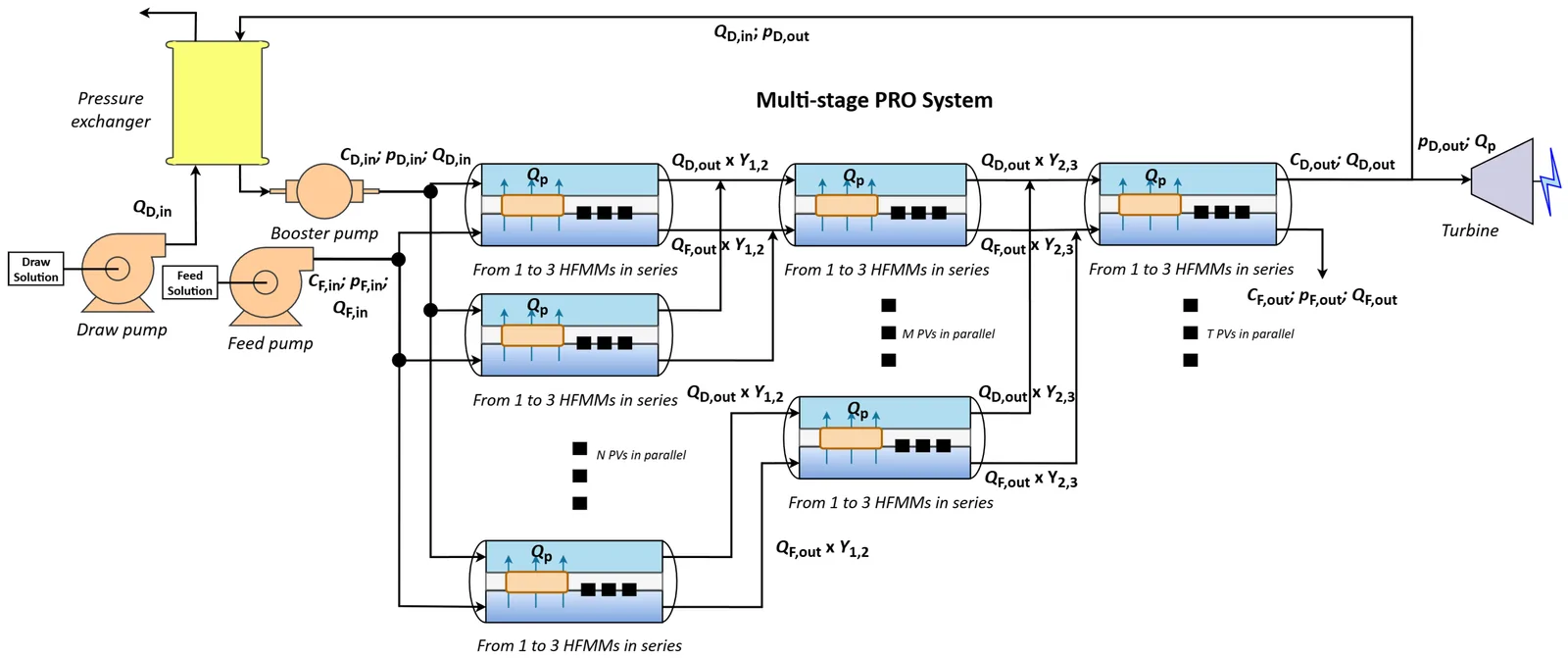
Diagram of a generic multistage PRO system with more than one PV in every stage, and more than one HFMM in every PV.

To estimate the ${\mathrm{SEG}}_{\mathrm{net}}$ that can be obtained from a single-stage, or even multistage, full-scale PRO system, the specific enthalpy (*h*) at the inlet and outlet of the hydraulic turbine and pumps needs to be known. Other devices, such as draw and feed pumps, ERDs (pressure exchanger and booster pump) and turbines were considered as part of the process. Figure 1 shows a flow diagram of the simulated PRO plant, considering these mentioned devices. In this study, a multistage full-scale PRO system is proposed; therefore, there are a number of PVs in parallel at each stage. The output flows from each stage are summed before entering the next stage, this is why the $Q_{D,\mathrm{out}}$ and $Q_{F,\mathrm{out}}$ are multiplied by a coefficient $Y_{12}$ (which refers to the parallel PVs in the first stage) or $Y_{23}$ (which refers to the parallel PVs in the second stage). Figure 1 also shows the direction followed by the draw and feed streams (PRO operating in co-current). Flow fractions $Y_{12}$ and $Y_{23}$ can be calculated as follows:

(9) $$Y_{12}=\frac{N_{\mathrm{PV},1}}{N_{\mathrm{PV},2}}$$

(10) $$Y_{23}=\frac{N_{\mathrm{PV},2}}{N_{\mathrm{PV},3}}$$

where $N_{\mathrm{PV}}$ is the number of PVs in a stage. The power (*P*) of the pumps, ERD and turbine were considered in this study. Based on the PRO system results, the power in the hydraulic turbine ($P_{\mathrm{TB}}$), draw, booster and feed pumps ($P_{\mathrm{DP}}$, $P_{\mathrm{BP}}$ and $P_{\mathrm{FP}}$, respectively) were calculated through the equations shown in Appendix A.2 of Appendix A.

(11) $$P_{\mathrm{net}}=P_{\mathrm{TB}}-P_{\mathrm{DP}}-P_{\mathrm{BP}}-P_{\mathrm{FP}}$$

where the efficiency (η) of the hydraulic turbine is considered 85% in this case and η of the three pumps is assumed as 80%. $P_{\mathrm{DP}}$, $P_{\mathrm{BP}}$ and $P_{\mathrm{FP}}$ will remain the same as in the single-stage process, since pumping between stages has not been taken into account.

(12) $${\mathrm{SEG}}_{\mathrm{net}}=\frac{P_{\mathrm{net}}}{Q_{p}}$$

The SEG was used to obtain specific measurements of energy generation per m^3^ of $Q_{p}$, allowing configurations with different feed-to-draw flow ratios and different numbers of stages to be compared on a common basis.

## 3. Results and Discussion

### 3.1. Impact of the Concentration Gradient on ${\mathrm{SEG}}_{\mathrm{Net}}$

The two draw concentrations, $C_{D,\mathrm{in}}$ = 30 g L^−1^ and 60 g L^−1^, were compared under their respective optimal first-stage input conditions (Table 4, Table 5, Table 6 and Table 7). Doubling the draw concentration increased the recoverable energy by a factor of more than three. For the three-stage configuration with a single HFMM in the third stage, ${\mathrm{SEG}}_{\mathrm{net}}$ rose from 129.87 Wh m^−3^ at 30 g L^−1^ to 421.48 Wh m^−3^ at 60 g L^−1^, while the $P_{\mathrm{TB}}$ from 1774.58 W to 4426.31 W ($Y_{12}$ was 1.2 and $Y_{23}$ was 3 for 30 g L^−1^, while $Y_{12}$ was 1.2 and $Y_{23}$ was 2.6 for 60 g L^−1^). The effect was even more pronounced in the two-stage configuration, where the system shifted from being a net energy consumer (−27.94 Wh m^−3^) to delivering 248.32 Wh m^−3^ ($Y_{12}$ = 1.2 for both 30 and 60 g L^−1^).

The energy consumed by the pumping system scales essentially with the circulated $Q_{D,\mathrm{in}}$ and $Q_{F,\mathrm{in}}$ (and the applied $p_{D,\mathrm{in}}$ through the ERD), whereas the energy delivered by the turbine scales with the product of the $Q_{p}$ and the available $p_{\mathrm{out}}$, although both depend on on $p_{D,\mathrm{in}}$. A stronger concentration gradient therefore both raises the driving force and allows the hydraulic penalty of the system to be amortized over a larger energy output. Consistently, the optimal first-stage $p_{D,\mathrm{in}}$ almost doubled when $C_{D,\mathrm{in}}$ was doubled, from 15 to 29.5 bar in the three-stage system and from 10 to 27.5 bar in the two-stage system, and the optimal $Q_{F,\mathrm{in}}$ increased from 5.5 to 7 m^3^ h^−1^ and from 3.5 to 5.5 m^3^ h^−1^, respectively, while $Q_{D,\mathrm{in}}$ remained at 3 m^3^ h^−1^ in all cases.

Al-Zainati et al. [39] reported an ${\mathrm{SEG}}_{\mathrm{net}}$ of 263 Wh m^−3^ for their best three-stage configuration and 279 Wh m^−3^ for a four-stage one, using a Dead Sea (DS) of 292.2 g L^−1^ against an RO brine FS of 70.13 g L^−1^. These values cannot be compared directly with the 415 to 421 Wh m^−3^ obtained here, since the two studies employ different normalization bases: $SEG_{\mathrm{net}}$ refers to the $Q_{p}$ in the present work and to the total inlet flow rate in [39]. A detailed comparison of the modeling details of both studies is given in Table 8.

The negative ${\mathrm{SEG}}_{\mathrm{net}}$ values obtained at 29.5 g L^−1^ with two stages (Table 6) deserve attention, since they identify an operational limit rather than a modeling artifact: with such a moderate gradient, two stages are not sufficient for the harvested osmotic energy to pay back the pumping demand of a full-scale system, regardless of how many HFMMs are placed in series in the second stage. This threshold behavior is one of the central practical outcomes of the present study.

**Figure 2:**
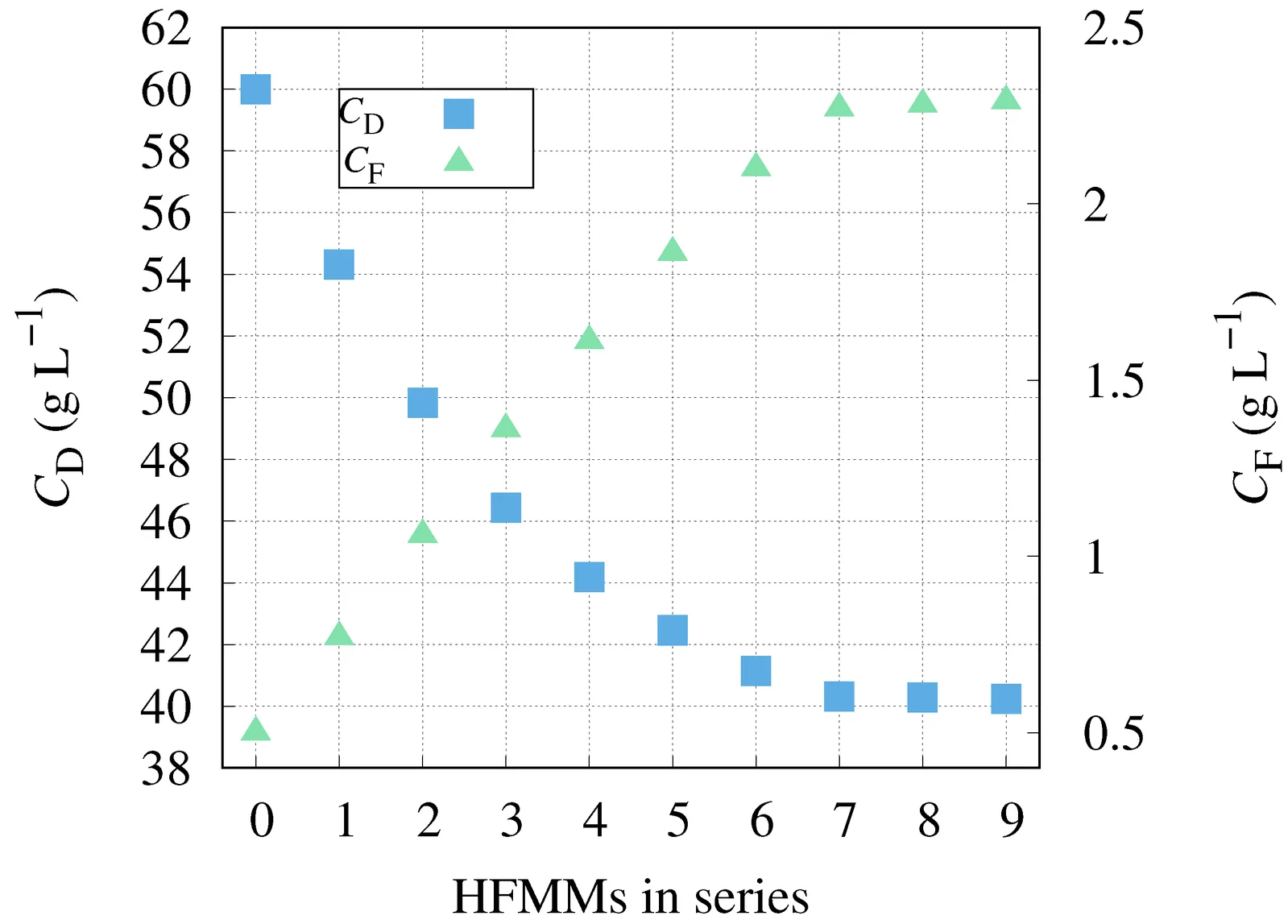
Dilution of $C_{D}$ and concentration of $C_{F}$ when the number of HFMMs arranged in series is increased, for the conditions that maximize the ${\mathrm{SEG}}_{\mathrm{net}}$ with three stages, $Y_{12}$ = 1.2 and $Y_{23}$ = 2.5.

The reason that the concentration gradient was depleted so quickly along a series arrangement is shown in Figure 2. Starting from $C_{D,\mathrm{in}}$ = 60 g L^−1^, $C_{D}$ fell steeply over the first modules and then leveled off at approximately 40 g L^−1^ beyond six or seven HFMMs in series, while $C_{F}$ rose from about 0.5 to roughly 2.3 g L^−1^ and stabilized as well. Once both streams approached this asymptote, the local Δπ across the membrane was no longer able to sustain a significant $J_{p}$, and additional membrane area placed in series contributed hydraulic losses rather than energy. The main cause of this behavior is that the applied hydraulic pressure remains high despite the losses along the series, whereas Δπ decreases module after module as the DS is diluted and the FS is concentrated. The asymptotic values reached in Figure 2 correspond to a bulk Δπ of approximately 29 bar, close to the applied Δp, so that the net driving force (Δπ−Δp) in Equation (1) approaches zero and the system tends towards osmotic–hydraulic equilibrium. The remaining difference is absorbed by the ECP and ICP effects, which make the Δπ available at the membrane surface lower than the bulk value. In addition, $J_{s}$ does not vanish as $J_{p}$ approaches zero, so salt continues to be transferred from the DS to the FS without any associated energy recovery. Consequently, each additional HFMM placed in series still contributes its axial pressure loss on both sides, reducing the $p_{D,\mathrm{out}}$ available at the turbine, while producing a negligible amount of permeate.

### 3.2. Impact of the Number of Stages on ${\mathrm{SEG}}_{\mathrm{Net}}$

The benefit of adding the third stage was quantified by comparing the optimal two- and three-stage systems under identical $C_{D}$ and identical numbers of HFMMs in series in the last stage (Table 4, Table 5, Table 6 and Table 7). At the salinity gradient of 59.5 g L^−1^, the third stage increased ${\mathrm{SEG}}_{\mathrm{net}}$ from 248.32 to 421.48 Wh m^−3^ for *n* = 1 (+69.7%), from 270.61 to 418.50 Wh m^−3^ for *n* = 2 (+54.7%) and from 286.60 to 415.07 Wh m^−3^ for *n* = 3 (+44.8%). $Y_{23}$ was 2.6 for *n* = 1, and 2.5 for *n* = 2 or 3. At 29.5 g L^−1^, the improvement was qualitative rather than quantitative, since the third stage turned a net-consuming system into a net-producing one for every value of *n*. The marginal contribution of the third stage decreased as the last stage of the two-stage system was lengthened, which was expected: a longer second stage already extracted part of the energy that the third stage would otherwise recover. Also, the number of modules arranged in series in the last stage acted in opposite directions in the two configurations. In the two-stage system, increasing *n* from 1 to 3 raised ${\mathrm{SEG}}_{\mathrm{net}}$ from 248.32 to 286.6 Wh m^−3^ at 59.5 g L^−1^ and from −27.94 to −1.52 Wh m^−3^ at 29.5 g L^−1^ ($Y_{12}$ = 1.2 in every case), whereas in the three-stage system, it produced a slight but systematic penalty, from 129.87 to 127.05 Wh m^−3^ and from 421.48 to 415.07 Wh m^−3^.

The relative gain obtained in the present work by adding a third stage (between 44.8% and 69.7%, depending on *n* of the third stage) is considerably larger than the 23.69–26.10% improvement reported by Al-Zainati et al. [39] for a three-stage system relative to a single-stage one. The discrepancy was attributable to the different design philosophy: in Al-Zainati’s study, the total number of membrane modules was fixed at four and redistributed among the stages, so that adding a stage did not add membrane area, whereas, in the present full-scale configuration, each stage incorporated its own PVs. Also, Al-Zainati et al. [39] found variations of up to 11.98% in the net specific energy between dual-stage configurations, and concluded that placing more modules in the last stage was advantageous. The present results agree with that conclusion for the two-stage system, but show the opposite trend once a third stage is introduced, which suggests that the benefit of lengthening the final stage vanishes as soon as the number of stages is sufficient to keep the driving force restored.

Table 8 summarizes the modeling details of both studies. Beyond the normalization basis discussed in Section 3.1, three aspects account for the differences between the results. First, the reference case against which the benefit of staging is measured is not the same: the gains in the present work correspond to the addition of a third stage to a two-stage system, whereas those of [39] correspond to a three-stage system relative to a single-stage one, so that the baseline adopted in the present work is more demanding. Second, the process configurations differ, since a fresh FS is supplied at the inlet of every stage in [39], whereas the FS traverses the whole system in the present study and the pumping energy is charged exclusively to the first stage. Third, the salinity gradient regimes are not comparable. The transport phenomena included in both models, on the other hand, are equivalent, which indicates that the differences arise from the system configuration and the evaluation criteria rather than from the underlying description of the process.

A broader benchmark against the literature is hindered by the fact that many published works report gross PD rather than net energy generation (Table 1). Restricting the comparison to those studies in which the pumping demand was discounted, one of the most informative reference is [46], which employed the same HFMM, the same mathematical model and the same salinity gradient of 59.5 g L^−1^ in a single-stage full-scale configuration, so that the contribution of staging can be isolated from any other effect. Although $SEG_{\mathrm{net}}$ was not reported in the work, it can be calculated from the $P_{\mathrm{net}}$, *R* and $Q_{F,\mathrm{in}}$ values given therein, yielding approximately 208 Wh m^−3^. The two-stage system analyzed here delivered 248.32 Wh m^−3^, a 19% improvement, while the three-stage system reached 421.48 Wh m^−3^, which represents an increase of 102% with respect to the single-stage configuration (each system operating at its own optimal conditions). The marginal contribution of the third stage is thus considerably larger than that of the second, which supports one of the conclusions of this study: at moderate salinity gradients, the energy still contained in the DS after two stages is sufficient to justify a third one.

**Figure 3:**
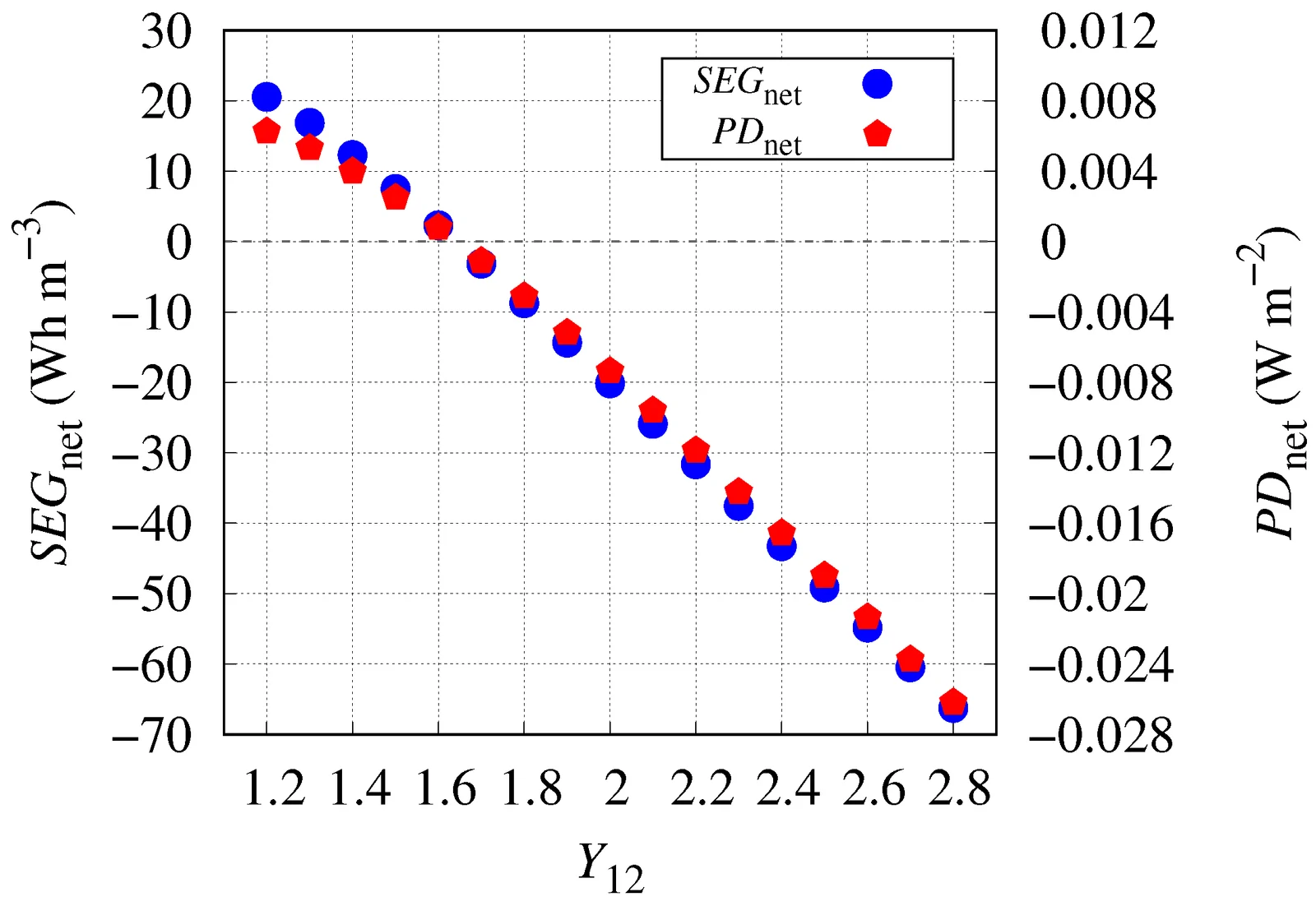
${\mathrm{SEG}}_{\mathrm{net}}$ and ${\mathrm{PD}}_{\mathrm{net}}$ trends as $Y_{12}$ increases, considering a two-stage system with 3 HFMMs per PV, for the conditions of $C_{D,\mathrm{in}}$ = 60 g L^−1^, $p_{D,\mathrm{in}}$ = 37.5 bar, $Q_{D,\mathrm{in}}$ = 6 m^3^ h^−1^ and $Q_{F,\mathrm{in}}$ = 6 m^3^ h^−1^.

The distribution of PVs between stages is decisive. Figure 3 shows ${\mathrm{SEG}}_{\mathrm{net}}$ and ${\mathrm{PD}}_{\mathrm{net}}$ as a function of $Y_{12}$. Both magnitudes decreased monotonically and almost linearly over the whole range explored, from approximately 21 Wh m^−3^ at $Y_{12}$ = 1.2 to about −66 Wh m^−3^ at $Y_{12}$ = 2.8, with the system ceasing to deliver net energy beyond $Y_{12}\approx$ 1.7. Tapered arrays, which are standard practice in RO, are therefore counterproductive in PRO. In an RO train, the concentrate flow decreases from stage to stage as the permeate is withdrawn, which justifies reducing the number of PVs downstream in order to keep the cross-flow velocity within its operating window. In a PRO train, the opposite occurs: the draw stream gains the permeated water and leaves each stage with a larger $Q_{D}$ than it entered with. Forcing that increased *Q* through fewer PVs raises the cross-flow velocity and the associated Δp, and the additional pumping demand is not compensated by any gain in osmotic driving force. Arrangements such as 3:1 are consequently penalized. It should be noted that the explored range was limited to $Y_{12}\geq 1.2$, so configurations in which the second stage is equipped with at least as many PVs as the first were not simulated.

### 3.3. Efficiency of the Multistage PRO System

**Figure 4:**
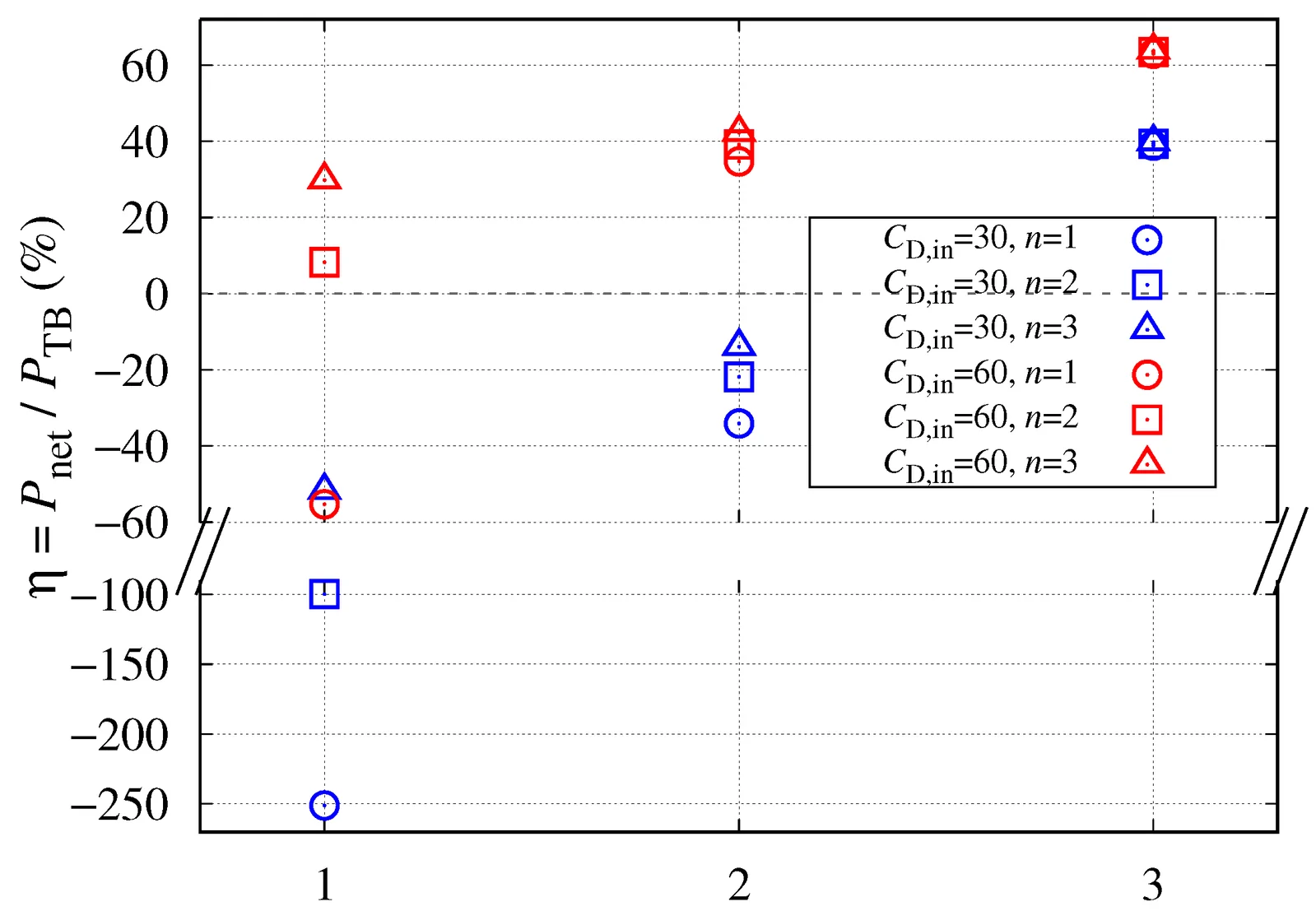
Maximum efficiency achieved per stage when 1, 2 or 3 HFMMs arranged in series per PV are considered in all the stages, for the input conditions shown in Table 4 and Table 5.

The efficiency of each stage is shown in Figure 4 for both $C_{D}$ and for 1, 2 and 3 HFMMs in series per PV in all stages. The first stage was a net energy consumer in almost every case: at 30 g L^−1^, its efficiency was strongly negative and improved markedly with *n*, from approximately −250% with a single membrane module to about −55% with three, and at 60 g L^−1^, it only became positive when two or more modules were arranged in series per PV. This reflected the fact that the pressurization of the draw stream was charged entirely to the first stage, while the $J_{p}$ it generated was limited by the short residence time of a single module. The efficiency then recovered rapidly along the cascade. In the second stage, the 60 g L^−1^ system already operated at around 35–42%, while the 30 g L^−1^ system remained slightly negative. In the third stage, both concentrations delivered positive efficiencies of the order of 38–39% at 30 g L^−1^ and 63–64% at 60 g L^−1^, and, importantly, the results for *n* = 1, 2 and 3 converged almost completely. The number of HFMMs per PV is therefore a critical design variable in the first stage and an almost irrelevant one in the last, which is a useful issue for capital cost allocation in a full-scale system.

A stage-resolved energy balance of this kind has not been reported for multistage PRO systems. Al-Zainati et al. [39] resolved the $Q_{p}$, $J_{p}$, Δπ and Δp stage by stage, but evaluated the ${\mathrm{SEG}}_{\mathrm{net}}$ only at system level, reporting single values of between 209 and 279 Wh m^−3^ for the whole cascade. Such an aggregate metric conceals the fact that the first stage of a full-scale system operates as a net energy consumer, and that the profitability of the process rests entirely on the last stages of the system. The distinction is not merely descriptive: it identifies the first stage as the element where the number of modules per PV must be optimized, and it explains why configurations that appear equivalent in terms of aggregate energy output may differ substantially in the way that energy is produced and consumed along the cascade.

**Figure 5:**
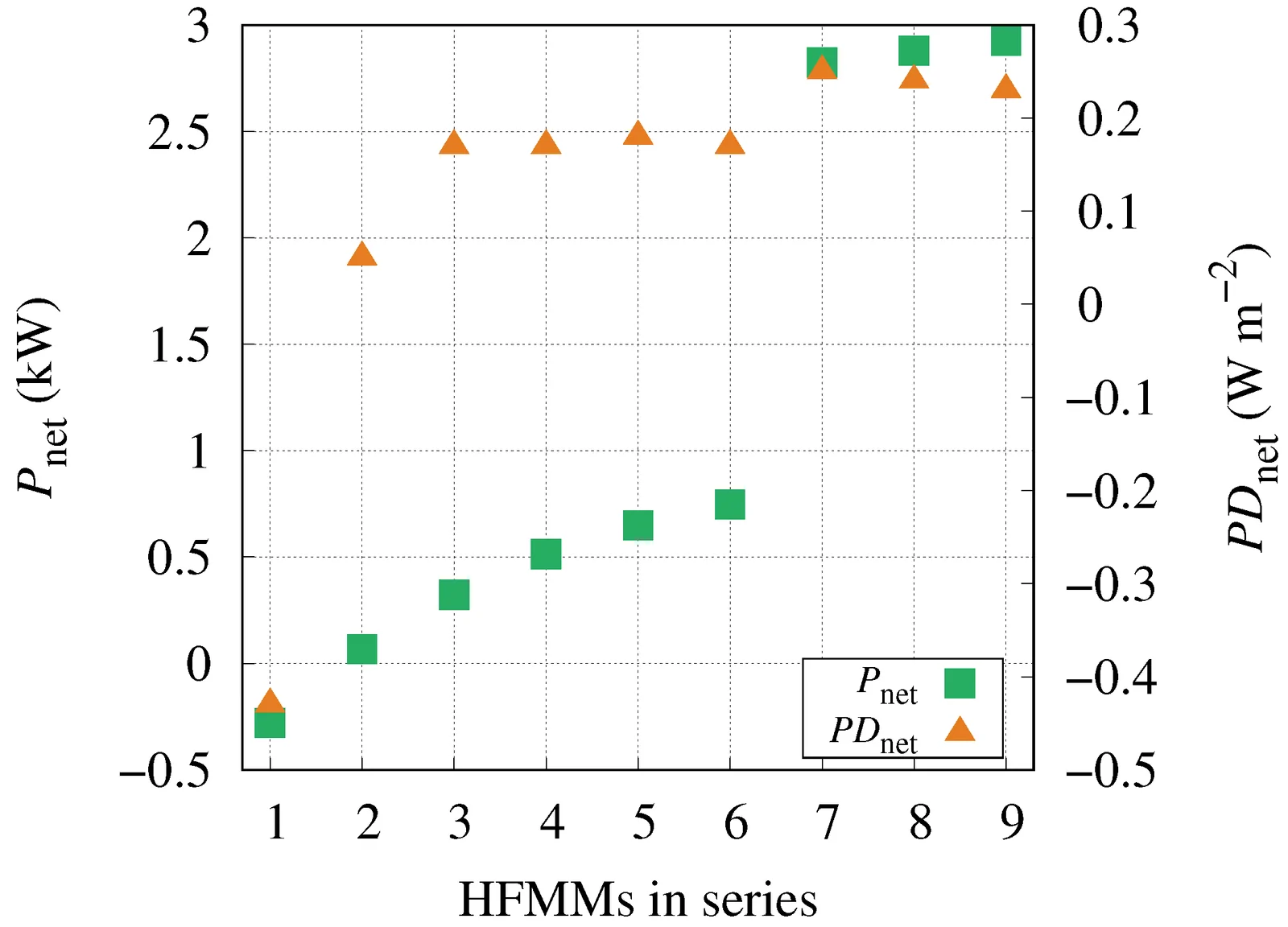
$P_{\mathrm{net}}$ and ${\mathrm{PD}}_{\mathrm{net}}$ trends as the number of HFMMs arranged in series increases, for the conditions of $C_{D,\mathrm{in}}$ = 60 g L^−1^, $p_{D,\mathrm{in}}$ = 29.5 bar, $Q_{D,\mathrm{in}}$ = 3 m^3^ h^−1^ and $Q_{F,\mathrm{in}}$ = 7.5 m^3^ h^−1^.

The same behavior was observed at module level in Figure 5, obtained for $C_{D,\mathrm{in}}$ = 60 g L^−1^ and $p_{D,\mathrm{in}}$ = 29.5 bar. A single HFMM operating alone yielded negative $P_{\mathrm{net}}$, since the whole pumping demand is charged to the inlet of the train while the permeate produced by one module is insufficient to compensate it. $P_{\mathrm{net}}$ became positive from the second module onwards, and continued to increase, while ${\mathrm{PD}}_{\mathrm{net}}$ rose steeply between the first and third HFMMs and then flattened out. This behavior follows from the definition of ${\mathrm{PD}}_{\mathrm{net}}$, in which the membrane area in the denominator grows linearly with the number of membrane modules in series. Over the first modules, the concentration gradient remained close to its inlet value (Figure 2), so that Δπ−Δp was large and each additional module contributed a substantial amount of permeate, making $P_{\mathrm{net}}$ grew faster than the accumulated $S_{m}$. As the DS is diluted and the FS is concentrated, the marginal permeate production of each additional module decreases while the area keeps growing at the same rate, so that both terms of the ratio evolve at comparable rates and ${\mathrm{PD}}_{\mathrm{net}}$ levels off. Beyond that point, the additional area contributed pressure drop rather than driving force, in agreement with the concentration profiles of Figure 2, which is consistent with the performance-limiting effects described for PRO systems [50]. The knee of this curve therefore identifies the number of HFMMs in series beyond which additional membrane area no longer improves the energy recovered per unit of installed area, a criterion that has not been reported for full-scale multistage PRO systems.

## 4. Conclusions

The ${\mathrm{SEG}}_{\mathrm{net}}$ of a full-scale multistage PRO system was evaluated for the salinity gradients of 29.5 and 59.5 g L^−1^ using HFMMs, where the salinity gradient was found to govern the viability of the process far more strongly than any other variable. Doubling $C_{D}$ more than triples the recoverable energy, from 129.87 to 421.48 Wh m^−3^ in the three-stage configuration, and simultaneously raises the optimal first-stage value of $p_{D,\mathrm{in}}$ from 15 to 29.5 bar. The number of stages proved equally decisive: adding a third stage increases ${\mathrm{SEG}}_{\mathrm{net}}$ by between 44.8% and 69.7% at 59.5 g L^−1^ and, at 29.5 g L^−1^, transforms a system that consumes more energy than it produces into a net energy producer. Two stages are therefore insufficient to recover the pumping demand of a full-scale system operating at moderate salinity gradients, regardless of the membrane area installed in the second stage.

The results also identified where additional membrane area ceases to be profitable. $C_{D}$ levels off at approximately 40 g L^−1^ beyond six or seven HFMMs arranged in series, so that further modules contribute hydraulic losses rather than driving force; consistently, lengthening the last stage improves ${\mathrm{SEG}}_{\mathrm{net}}$ in the two-stage system but slightly penalizes it in the three-stage one. The distribution of PVs between stages follows the opposite logic to RO practice: since the DS gains volume as it advances, tapered arrangements such as 3:1 are counterproductive, and the second stage should be equipped with at least as many PVs as the first. The stage-resolved efficiency shows that the first stage operates as a net energy consumer while the third reaches 63–64% at 59.5 g L^−1^, which locates the number of membranes per PV as a critical design variable in the first stage and an almost irrelevant one in the last stage. These results should nevertheless be interpreted within the limitations of the model employed: the simulations assumed steady-state operation with a clean membrane, so that neither fouling nor the long-term flux decline associated with it were accounted for, and the fouling factor was kept at unity throughout; the ECP and ICP effects were included in the transport equations, but they were evaluated from average values per HFMM, so that their local variation within each module was not resolved. The optimal operating conditions reported in the present work should therefore be regarded as an upper bound of the performance attainable by a full-scale multistage PRO system, and as a basis for the comparison between configurations, rather than as design values directly transferable to an industrial-scale plant. A distributed-parameter model of the membrane module, the incorporation of fouling and long-term operation, and the experimental validation at pilot scale, together with a techno-economic assessment of the additional PVs demanded by each stage, remain necessary steps before multistage PRO can be proposed for industrial deployment.

## Figures and Tables

**Table 1:** Summary of PRO studies.

Reference	Configuration	SG ($g\;L^{-1}$)	PD ($W\;m^{-2}$)	Axial Pressure Drop	Axial Concentration Variation	Net Energy
Chou et al. [20]	Single-stage/1 HFMM	58.4	20.9	No	No	No
Sun and Chung [21]	Single-stage/1 HFMM	58.5	7.63	Yes	Yes	No
Achilli et al. [22]	Single-stage/1 SWMM	34.8	1.15	Yes	Yes	Yes
Attarde et al. [23]	Single-stage/1 SWMM	59	3.29	No	No	No
Lee et al. [24]	Single-stage/1 SWMM	34.9	1.77	Yes	Yes	No
O’Toole et al. [25]	Two-stage (3:1)	SW/FW	0.84	Yes	Yes	Yes
O’Toole et al. [25]	Single-stage	SW/FW	0.47	Yes	Yes	Yes
Higa et al. [26]	Single-stage/1 HFMM	29.25	0.14	Yes	Yes	No
Kishimoto et al. [27]	Single-stage/1 HFMM	35	1.6	No	Yes	No
Benjamin et al. [28]	Single-stage	26–66	16.7	Yes	Yes	Yes
Aseffa et al. [29]	Single-stage/1 HFMM	35.66	0.45	Yes	Yes	Yes
Matsuyama et al. [30]	Single-stage/1 HFMM	34.8	2.8	Yes	Yes	Yes
Al-Zainati et al. [31]	Dual-stage	257.4	–	Yes	Yes	No
Matta et al. [32]	Single-stage	125.2	9.8	Yes	No	No
Ruiz-García et al. [33]	Single-stage/1–8 SWMMs	49.5	3.46	Yes	Yes	No
Low et al. [34]	Lab-scale HFMM	58.5	9.1	No	No	No
Low et al. [34]	Pilot-scale HFMM	58.5	2.5	No	No	No
Abdelkader et al. [35]	Single-stage	59.4	4.0	Yes	Yes	No
Ng et al. [36]	Single-stage	RO brine	15.0	Yes	Yes	Yes
Ruiz-García et al. [37]	Single-stage/1–8 SWMM	179.5	14.31	Yes	Yes	No
Yagnambhatt et al. [38]	Flat-sheet	70	2.0	Yes	No	Yes
Al-Zainati et al. [39]	Multistage HFMM	291.0	15.0	Yes	Yes	Yes
Ruiz-García [40]	Single-stage/1–8 SWMM	59.5–179.5	0.73–3.1	Yes	Yes	Yes
AL-Musawi et al. [41]	Single-stage/1 SWMM	51.8	2.83	–	–	Yes
AlZainati et al. [42]	Single-/multistage	3.3–207.5	0.02–120	No	No	No
Alnajdi et al. [43]	Single-stage/1 HFMM	70	1.5	Yes	Yes	Yes
Kim et al. [44]	Single-stage/1 SWMM	77.2	9.8	–	Yes	No
Touati et al. [45]	Single-stage	70.1	–	Yes	–	Yes
Suárez-Alfonso et al. [46]	Single-stage/1–3 HFMMs	59.5–179.5	0.28–2.56	Yes	Yes	Yes

**Table 2:** Simulation parameters and operating ranges.

Parameter	Range or Value
FF	1.0
*T* (°C)	25
$C_{D,\mathrm{in}}$ (g L^−1^)	30–60
$\Delta C_{D,\mathrm{in}}$	30
$C_{F,\mathrm{in}}$ (g L^−1^)	0.5
$p_{D,\mathrm{in}}$ (bar)	3–50
$\Delta p_{D,\mathrm{in}}$	0.5
$p_{F,\mathrm{in}}$ (bar)	2
$Q_{D,\mathrm{in}}$ (m^3^ h^−1^)	3–16
$\Delta Q_{D,\mathrm{in}}$	0.5
$Q_{F,\mathrm{in}}$ (m^3^ h^−1^)	3–16
$\Delta Q_{F,\mathrm{in}}$	0.5
*Y* from stage 1 to stage 2	1.2–3
*Y* from stage 2 to stage 3	1.2–3

**Table 3:** Parameters of the 10-inch PRO HFMM.

Parameter	Value
$A_{0}$ (m Pa^−1^ s^−1^)	3.1 × $10^{-13}$
*B* (m s^−1^)	1.5 × $10^{-8}$
$S_{f}$ (μm)	460
$S_{m}$ (m^2^)	650
*L* (m)	0.68
$H_{D}$ (m)	1.1 × $10^{-3}$
$H_{F}$ (m)	1.5 × $10^{-3}$
$d_{i}$ (m)	105 × $10^{-6}$
$R_{i}$ (m)	1.27 × $10^{-2}$
$R_{o}$ (m)	5.33 × $10^{-2}$
$\varepsilon_{D}$	0.4
$\varepsilon_{F}$	1.0

**Table 4:** Input conditions (first stage) that maximize ${\mathrm{SEG}}_{\mathrm{net}}$ with three stages, salinity gradient of 29.5 g L^−1^, and the corresponding results obtained when *n* = 1, 2 or 3 HFMMs in series are considered in the third stage.

Input Conditions (1st Stage)				*n*	Results (3rd Stage)		
$C_{D,\mathrm{in}}$ (g L^−1^)	$p_{D,\mathrm{in}}$ (bar)	$Q_{D,\mathrm{in}}$ (m^3^ h^−1^)	$Q_{F,\mathrm{in}}$ (m^3^ h^−1^)		R (%)	$P_{\mathrm{TB}}$ (W)	${\mathrm{SEG}}_{\mathrm{net}}$ (Wh m^−3^)
30	15	3	5.5	1	26.78	1774.58	129.87
				2	27.55	1784.59	128.57
				3	28.32	1793.56	127.05

**Table 5:** Input conditions (first stage) that maximize ${\mathrm{SEG}}_{\mathrm{net}}$ with three stages, salinity gradient of 59.5 g L^−1^, and the corresponding results obtained when *n* = 1, 2 or 3 HFMMs in series are considered in the third stage.

Input Conditions (1st Stage)				*n*	Results (3rd Stage)		
$C_{D,\mathrm{in}}$ (g L^−1^)	$p_{D,\mathrm{in}}$ (bar)	$Q_{D,\mathrm{in}}$ (m^3^ h^−1^)	$Q_{F,\mathrm{in}}$ (m^3^ h^−1^)		R (%)	$P_{\mathrm{TB}}$ (W)	${\mathrm{SEG}}_{\mathrm{net}}$ (Wh m^−3^)
60	29.5	3	7	1	30.29	4426.31	421.48
60	29.5	3	7.5	2	30.58	4538.93	418.5
60	29.5	3	7.5	3	31.34	4587.87	415.07

**Table 6:** Input conditions (first stage) that maximize ${\mathrm{SEG}}_{\mathrm{net}}$ with two stages, salinity gradient of 29.5 g L^−1^, and the corresponding results obtained when *n* = 1, 2 or 3 HFMMs in series are considered in the second stage.

Input Conditions (1st Stage)				*n*	Results (2nd Stage)		
$C_{D,\mathrm{in}}$ (g L^−1^)	$p_{D,\mathrm{in}}$ (bar)	$Q_{D,\mathrm{in}}$ (m^3^ h^−1^)	$Q_{F,\mathrm{in}}$ (m^3^ h^−1^)		R (%)	$P_{\mathrm{TB}}$ (W)	${\mathrm{SEG}}_{\mathrm{net}}$ (Wh m^−3^)
30	10	3	3.5	1	42.68	409.59	−27.94
30	10.5	3	3.5	2	44.3	442.82	−12.64
30	11	3	4	3	33.35	514.35	−1.52

**Table 7:** Input conditions (first stage) that maximize ${\mathrm{SEG}}_{\mathrm{net}}$ with two stages, salinity gradient of 59.5 g L^−1^, and the corresponding results obtained when *n* = 1, 2 or 3 HFMMs in series are considered in the second stage.

Input Conditions (1st Stage)				*n*	Results (2nd Stage)		
$C_{D,\mathrm{in}}$ (g L^−1^)	$p_{D,\mathrm{in}}$ (bar)	$Q_{D,\mathrm{in}}$ (m^3^ h^−1^)	$Q_{F,\mathrm{in}}$ (m^3^ h^−1^)		R (%)	$P_{\mathrm{TB}}$ (W)	${\mathrm{SEG}}_{\mathrm{net}}$ (Wh m^−3^)
60	27.5	3	5.5	1	31.15	1312.58	248.32
60	28.5	3	6.5	2	30.17	1549.26	270.61
60	29	3	7.5	3	29.85	1789.16	286.6

**Table 8:** Comparison of the modeling details of the present work and [39].

Aspect	Present Work	Al-Zainati et al. [39]
Membrane module	HFMM, 650 m^2^	HFMM, 63 m^2^
Modules per system	1–3 in series per PV, several PVs in parallel per stage	4 in total
Effect of adding a stage	Membrane area is added	Fixed area redistributed
Feed supply	Single feed at the first stage	Fresh feed at every stage
Inter-stage pumping	Not considered	Additional pump and splitter
DS	NaCl, 30–60 g L^−1^	Dead Sea, 292.2 g L^−1^
FS	0.5 g L^−1^	RO brine, 70.13 g L^−1^
Inlet flow rates	Optimized, 3–16 m^3^ h^−1^	Fixed, $Q_{\mathrm{tot}}$ = 2 m^3^ h^−1^
PV distribution between stages	Optimized ($Y_{12}$, $Y_{23}$)	Not considered
Normalization basis	$Q_{p}$	$Q_{\mathrm{tot}}$
Stage-resolved energy balance	Yes	No

## Data Availability

The datasets generated during the present study consist of the outputs of the simulations described throughout the manuscript. The raw data supporting the conclusions of this article will be made available by the authors on request.

## Abbreviations

The following abbreviations are used in this manuscript:

**Acronyms**	
DS	draw solution
ECP	external concentration polarization
ERD	energy-recovery device
FS	feed solution
HF	hollow fiber
HFMM	hollow-fiber membrane module
ICP	internal concentration polarization
PRO	pressure-retarded osmosis
PV	pressure vessel
RO	reverse osmosis
SG	salinity gradient
SW	spiral-wound
SWMM	spiral-wound membrane module
**Variables**	
$\dot{m}$	mass flow (kg s^−1^)
*A*	water permeability coefficient (m Pa^−1^ s^−1^)
$A_{0}$	initial value of *A* (m Pa^−1^ s^−1^)
*B*	solute permeability coefficient (m s^−1^)
CF	concentration factor
*C*	concentration (g L^−1^ or mol L^−1^)
DF	dilution factor
*D*	solute diffusivity (m^2^ s^−1^)
$D_{i}$	inner diameter of the fiber bundle (m)
$d_{h}$	hydraulic diameter of feed channel (m)
$d_{i}$	inner fiber diameter (m)
FF	fouling factor
*H*	spacer height (m)
*h*	specific enthalpy (J kg^−1^)
*J*	flux per unit area (m^3^ m^−2^ s^−1^)
$J_{s}$	solute flux per unit area (g m^−2^ s^−1^)
*K*	solute resistivity (s m^−1^)
*k*	mass transfer coefficient
*L*	length of the HFMM (m)
$N_{\mathrm{PV}}$	number of PVs in a stage
*n*	number of elements in series
PD	power density (W m^−2^)
*P*	power (W)
*p*	pressure (Pa or bar)
*Q*	flow rate (m^3^ h^−1^ or m^3^ s^−1^)
*R*	flux recovery percentage (%)
$R_{i}$	inner radius of the fiber bundle (m)
$R_{o}$	outer radius of the fiber bundle (m)
Re	Reynolds number
Sc	Schmidt number
$S_{f}$	structural parameter (μm)
Sh	Sherwood number
$S_{m}$	membrane surface (m^2^)
TCF	temperature correction factor
*T*	temperature (°C or K)
*v*	velocity (m s^−1^)
*Y*	flow fraction
**Greek letters**	
Δπ	osmotic pressure gradient (Pa or bar)
Δp	pressure gradient (Pa or bar)
η	performance
μ	dynamic viscosity (kg m^−1^ s)
π	osmotic pressure (Pa or bar)
ρ	density (kg m^−3^)
ε	porosity in feed channel
ϑ	specific volume (m^3^ kg^−1^)
**Subscripts**	
av	average
BP	booster pump
D	draw
DP	draw pump
F	feed
FP	feed pump
in	input
m	membrane
out	output
p	permeate
TB	turbine

## Appendix A. Equations of the PRO Mathematical Model

### Appendix A.1. Transport Phenomenon

The equations needed to calculate the different variables for Equation (1) and then for Equations (2)–(4) are shown below:

(A1) $$C_{D,m}=\left(C_{D,\mathrm{av}}+\frac{J_{s}}{J_{p}}\right)e^{\frac{-J_{p}}{k_{D}}}-\frac{J_{s}}{J_{p}}$$

(A2) $$C_{F,m}=\left(C_{F,\mathrm{av}}+\frac{J_{s}}{J_{p}}\right)e^{\frac{J_{p}}{k_{F}}}e^{KJ_{p}}-\frac{J_{s}}{J_{p}}$$

The different terms used in Equations (A1) and (A2) are then calculated as follows:

(A3) $$C_{D,\mathrm{av}}=0.5\left(C_{D,\mathrm{in}}+C_{D,\mathrm{out}}\right)$$

(A4) $$C_{F,\mathrm{av}}=0.5\left(C_{F,\mathrm{in}}+C_{F,\mathrm{out}}\right)$$

(A5) $$J_{s}=J_{p}\frac{B}{A\beta RT}\left(1+\frac{A\Delta p}{J_{p}}\right)$$

(A6) $$k_{D}=\frac{{\mathrm{Sh}}_{D}\;\cdot \;D_{D,\mathrm{av}}}{d_{h,D}}$$

(A7) $$k_{F}=\frac{{\mathrm{Sh}}_{F}\;\cdot \;D_{F,\mathrm{av}}}{d_{h,F}}$$

(A8) $$K=\frac{S_{f}}{D_{F,\mathrm{av}}}$$

where $C_{D,m}$ and $C_{F,m}$ are the membrane surface concentrations of the draw and feed sides considering ECP and ICP [51], $C_{D,\mathrm{av}}$ and $C_{F,\mathrm{av}}$ are the average concentrations of the draw and feed side, $J_{s}$ is the reverse solute flux, $k_{D}$ and $k_{F}$ are the mass transfer coefficients of the draw and feed sides, $C_{D,\mathrm{out}}$ is the output concentration in the draw side (and the same for $C_{F,\mathrm{out}}$ in the feed side), *K* is the solute resistivity and $S_{f}$ is the structural parameter of the HFMM. The structural parameter depends on the membrane support layer characteristics (i.e., thickness, tortuosity and porosity) and is used for the ICP quantification. The higher its value, the greater the ICP effect. β is the dimensionless van ’t Hoff factor for strong electrolytes (i.e., value of 2 for NaCl) [49], R is the gas constant (8.314 J mol^−1^ K^−1^) and *T* is the absolute temperature (in K) of the solution. Note that the proposed methodology can simulate PRO systems with different temperatures by considering *T* dependent equations for the diffusivity (*D*), density (ρ) and dynamic viscosity (μ). ${\mathrm{Sh}}_{D}$ and ${\mathrm{Sh}}_{F}$ are the Sherwood numbers for the draw (dilutive) and feed (concentrative), $D_{D}$ and $D_{F}$ are the diffusion coefficients of the DS and FS (using $C_{D,\mathrm{av}}$ and $C_{F,\mathrm{av}}$, respectively, in Equation (A15)), and $d_{h,D}$ and $d_{h,F}$ are the hydraulic diameters for the draw and feed side, respectively. Sh is a dimensionless number related to the ratio of convective to diffusive mass transport. Considering a laminar flow regime given the low cross-flow rate, as done in previous studies [33,52], ${\mathrm{Sh}}_{D}$ and ${\mathrm{Sh}}_{F}$ can be estimated with Equations (A9) and (A10), respectively:

(A9) $${\mathrm{Sh}}_{D}=0.048\;\cdot \;Re_{D}^{0.6}\;\cdot \;{\mathrm{Sc}}_{D}^{\frac{1}{3}}$$

(A10) $${\mathrm{Sh}}_{F}=0.048\;\cdot \;Re_{F}^{0.6}\;\cdot \;{\mathrm{Sc}}_{F}^{\frac{1}{3}}$$

(A11) $$Re_{D}=\frac{\rho_{D,\mathrm{av}}\;\cdot \;v_{D,\mathrm{av}}\;\cdot \;d_{h,D}}{\mu_{D,\mathrm{av}}}$$

(A12) $$Re_{F}=\frac{\rho_{F,\mathrm{av}}\;\cdot \;v_{F,\mathrm{av}}\;\cdot \;d_{h,F}}{\mu_{F,\mathrm{av}}}$$

(A13) $${\mathrm{Sc}}_{D}=\frac{\mu_{D,\mathrm{av}}}{\rho_{D,\mathrm{av}}\;\cdot \;D_{D}}$$

(A14) $${\mathrm{Sc}}_{F}=\frac{\mu_{F,\mathrm{av}}}{\rho_{F,\mathrm{av}}\;\cdot \;D_{F}}$$

(A15) $$D=-1.025\times 10^{-10}C+1.518\times 10^{-9}$$

(A16) $$d_{h,D}=\frac{4\varepsilon_{D}}{\frac{2}{H_{D}}+\left(1-\varepsilon_{D}\right)\;\cdot \;\frac{8}{H_{D}}}$$

(A17) $$d_{h,F}=\frac{4\varepsilon_{F}}{\frac{2}{H_{F}}+\left(1-\varepsilon_{F}\right)\;\cdot \;\frac{8}{H_{F}}}$$

where $Re_{D}$, $Re_{F}$, ${\mathrm{Sc}}_{D}$, ${\mathrm{Sc}}_{F}$, $\rho_{D,\mathrm{av}}$, $\rho_{F,\mathrm{av}}$, $\mu_{D,\mathrm{av}}$ and $\mu_{F,\mathrm{av}}$, $\varepsilon_{D}$, $\varepsilon_{F}$ are, for each side, respectively, the Reynolds number, the Schmidt number, the density of both solutions, the dynamic viscosity of both solutions and the porosity of the channels. Equations (A9) and (A10) correspond to the empirical correlation obtained in [52] from mass transfer measurements on complete hollow-fiber RO modules, which the authors reported as valid for both brackish-water- and seawater-type modules. Being an empirical correlation fitted at module level, its coefficients implicitly account for the irregular fiber arrangement and the associated flow maldistribution, rather than assuming an idealized boundary layer profile. The Reynolds numbers obtained in this study remain within the laminar range for which the correlation was established. The same correlation was applied to both sides of the membrane. Although the DS flows through the fiber lumen and the FS flows through the shell side, the correlation of [52] was derived at module level for HFMMs operating with both flow paths, and no equally validated side-specific correlation is available for the module considered here. This constitutes a simplification of the model, whose impact is limited by the predominance of the ICP discussed above. ρ and μ are calculated for each solution (DS and FS) through Equations (A18) and (A19) using $C_{D,\mathrm{av}}$ and $C_{F,\mathrm{av}}$ in mol L^−1^:

(A18) $$\rho =-1.047C^{2}+39.462C+997.970$$

(A19) $$\mu =0.001\times (0.012C^{2}+0.065C+0.985)$$

Summarizing Equation (1), the term Δp is obtained considering the pressure drop on both the draw and feed side.

(A20) $$\Delta p=p_{D,\mathrm{in}}-\frac{{\mathrm{PL}}_{D}}{2}-p_{F,\mathrm{in}}+\frac{{\mathrm{PL}}_{F}}{2}$$

In this study, the pressure loss in the draw side (${\mathrm{PL}}_{D}$) is calculated through the Hagen–Poiseuille equation, as proposed in previous works [53,54]:

(A21) $${\mathrm{PL}}_{D}=\frac{32\;\cdot \;\mu_{D,\mathrm{av}}\;\cdot \;v_{D,\mathrm{av}}}{d_{i}^{2}}L$$

The feed-side pressure loss (${\mathrm{PL}}_{F}$) is estimated similarly, but using both Hagen–Poiseuille and Ergun equations for pressure drop at the shell side [54].

(A22) $${\mathrm{PL}}_{F}=\frac{32\;\cdot \;\mu_{F,\mathrm{av}}\;\cdot \;v_{F,\mathrm{av}}}{D_{i}^{2}}L+\frac{150\;\cdot \;{\left(1-\varepsilon_{D}\right)}^{2}\;\cdot \;\mu_{F,\mathrm{av}}\;\cdot \;v_{F,\mathrm{av}}}{1.5^{2}\;\cdot \;\varepsilon_{D}^{3}\;\cdot \;d_{h,F}^{2}}\left(R_{o}-R_{i}\right)+\frac{1.75\;\cdot \;\left(1-\varepsilon_{D}\right)\;\cdot \;\rho_{F,\mathrm{av}}\;\cdot \;v_{F,\mathrm{av}}^{2}}{1.5\;\cdot \;\varepsilon_{D}^{3}\;\cdot \;d_{h,F}}\left(R_{o}-R_{i}\right)$$

where *L* is the length of the membrane module, $R_{o}$ the outer radius of the fiber bundle, $R_{i}$ the inner radius of the fiber bundle ($D_{i}$ is the diameter), and $d_{i}$ the inner fiber diameter.

$C_{D,\mathrm{out}}$ and $C_{F,\mathrm{out}}$ are affected by $Q_{p}$ and $J_{s}$, meaning that the DS is diluted and the FS is concentrated due to both $Q_{p}$ and $J_{s}$. The dilution factor and the concentration factor (DF and CF, respectively), as a result of $Q_{p}$, are defined in Equations (A23) and (A24), respectively.

(A23) $$\mathrm{DF}=\frac{C_{D,\mathrm{out}}}{C_{D,\mathrm{in}}}=\frac{1-Y_{m}}{Y_{m}}$$

(A24) $$\mathrm{CF}=\frac{C_{F,\mathrm{out}}}{C_{F,\mathrm{in}}}=\frac{1}{1-Y_{m}}$$

where $C_{D,\mathrm{out}}$ and $C_{F,\mathrm{out}}$ are the output concentrations as a result of only $Q_{p}$, and $Y_{m}$ is the recovery fraction of the HFMM ($Q_{p}$/$Q_{F,\mathrm{in}}$). The draw and feed-side mass fluxes (in kg s^−1^) are shown in the following Equations (A25) and (A26):

(A25) $$C_{D,\mathrm{out}}\;\cdot \;\left(Q_{D,\mathrm{in}}+Q_{p}\right)=C_{D,\mathrm{in}}\;\cdot \;\mathrm{DF}\;\cdot \;\left(Q_{D,\mathrm{in}}+Q_{p}\right)-J_{s}$$

(A26) $$C_{F,\mathrm{out}}\;\cdot \;\left(Q_{F,\mathrm{in}}-Q_{p}\right)=C_{F,\mathrm{in}}\;\cdot \;\mathrm{CF}\;\cdot \;\left(Q_{F,\mathrm{in}}-Q_{p}\right)+J_{s}$$

### Appendix A.2. Energy Equations

To estimate the net energy that can be generated from a single-stage full-scale PRO system, the specific enthalpy (*h*) at the inlet and outlet of the hydraulic turbine and pumps needs to be known. Devices including draw and feed pumps, an ERD (pressure exchanger and booster pump) and turbine form part of the process. Equations (A27)–(A29) are used to calculate *h* [55]. For non-atmospheric pressures ($p_{0}$), the specific enthalpy (h(T,p,C)) is estimated through Equation (A29). Based on the PRO system results, the power in the hydraulic turbine ($P_{\mathrm{TB}}$), draw, booster and feed pumps ($P_{\mathrm{DP}}$, $P_{\mathrm{BP}}$ and $P_{\mathrm{FP}}$, respectively) are calculated through Equations (A30)–(A37). The draw pump output pressure (input of the pressure exchanger) was 0.5 bar.

(A27) $$\begin{array}{c} h\left(T,p_{0},C\right)=h_{w}-C(b_{1}+b_{2}w_{s}+b_{3}w_{s}^{2}+b_{4}w_{s}^{3}+\\ b_{5}T+b_{6}T^{2}+b_{7}T^{3}+b_{8}w_{s}T+b_{9}w_{s}^{2}T+b_{10}w_{s}T^{2}) \end{array}$$

(A28) $$h_{w}=141.355+4202.070T-0.535T^{2}+0.004T^{3}$$

$$\begin{array}{c} b_{1}=-2.348\times 10^{4},b_{2}=3.152\times 10^{5},b_{3}=2.803\times 10^{6},\\ b_{4}=-1.446\times 10^{7},b_{5}=7.826\times 10^{3},b_{6}=-4.417\times 10^{1},\\ b_{7}=2.139\times 10^{-1},b_{8}=-1.991\times 10^{4},b_{9}=2.778\times 10^{4},\\ b_{10}=9.728\times 10^{1} \end{array}$$

(A29) $$h\left(T,p,C\right)=h\left(T,p_{0},C\right)+\vartheta \left(p-p_{0}\right)$$

where ϑ is the specific volume (the inverse of ρ). ϑ is determined for both the DS and FS using the ρ of the DS and FS at the inlet and outlet of the different devices.

(A30) $$P_{\mathrm{TB}}=\eta_{\mathrm{TB}}\;\cdot \;{\dot{m}}_{\mathrm{TB}}\;\cdot \;\left(h_{\mathrm{TB},\mathrm{in}}-h_{\mathrm{TB},\mathrm{out}}\right)$$

(A31) $${\dot{m}}_{\mathrm{TB}}=Q_{p}\;\cdot \;\rho_{D,\mathrm{out}}$$

(A32) $$P_{\mathrm{DP}}=\frac{{\dot{m}}_{\mathrm{DP}}\;\cdot \;\left(h_{\mathrm{DP},\mathrm{in}}-h_{\mathrm{DP},\mathrm{out}}\right)}{\eta_{\mathrm{DP}}}$$

(A33) $${\dot{m}}_{\mathrm{DP}}=Q_{D,\mathrm{in}}\;\cdot \;\rho_{D,\mathrm{in}}$$

(A34) $$P_{\mathrm{BP}}=\frac{{\dot{m}}_{\mathrm{BP}}\;\cdot \;\left(h_{\mathrm{BP},\mathrm{in}}-h_{\mathrm{BP},\mathrm{out}}\right)}{\eta_{\mathrm{BP}}}$$

(A35) $${\dot{m}}_{\mathrm{BP}}=Q_{D,\mathrm{in}}\;\cdot \;\rho_{D,\mathrm{in}}$$

(A36) $$P_{\mathrm{FP}}=\frac{{\dot{m}}_{\mathrm{FP}}\;\cdot \;\left(h_{\mathrm{FP},\mathrm{in}}-h_{\mathrm{FP},\mathrm{out}}\right)}{\eta_{\mathrm{FP}}}$$

(A37) $${\dot{m}}_{\mathrm{FP}}=Q_{F,\mathrm{in}}\;\cdot \;\rho_{F,\mathrm{in}}$$

(A38) $$P_{\mathrm{net}}=P_{\mathrm{TB}}-P_{\mathrm{DP}}-P_{\mathrm{BP}}-P_{\mathrm{FP}}$$

(A39) $$\mathrm{PD}=\frac{P_{\mathrm{TB}}}{nS_{m}}$$

(A40) $${\mathrm{PD}}_{\mathrm{net}}=\frac{P_{\mathrm{net}}}{nS_{m}}$$

where $\eta_{\mathrm{TB}}$ is the efficiency of the hydraulic turbine (85% was considered in this case) and the η of the three pumps is assumed as 80%. *n* is the number of HFMMs in series of the PRO system and will be affected by $Y_{12}$ and $Y_{23}$, as all the membranes from each stage have to be considered when calculating the PD of the PRO system.
